# What is the role of Swiss domestic cats in environmental contamination with *Echinococcus multilocularis* eggs?

**DOI:** 10.1186/s13071-023-05983-y

**Published:** 2023-10-09

**Authors:** Rebecca Furtado Jost, Norbert Müller, Nelson Marreros, Gastón Moré, Loic Antoine, Walter Basso, Caroline F. Frey

**Affiliations:** 1 Institute of Parasitology, Department of Infectious Diseases and Pathobiology, Vetsuisse Faculty, University of Bern, Länggassstrasse 122, 3012 Bern, Switzerland; 2 School of Agricultural, Forest and Food Sciences HAFL, Bern University of Applied Sciences, Länggasse 85, 3052 Zollikofen, Switzerland; 3 Boehringer-Ingelheim Animal Health, 29 Avenue Tony Garnier, 69007 Lyon, France

**Keywords:** Coprology, Copro-qPCR, Intestinal scraping technique, Fox tapeworm, *Taenia*, *Hydatigera*, *Mesocestoides*, *Toxocara cati*, *Toxoplasma gondii*, *Strongyloides*, Zoonoses

## Abstract

**Background:**

The role of the domestic cat as definitive host for *Echinococcus multilocularis* and thus in environmental contamination with eggs has not yet been entirely resolved. This study aimed to assess the prevalence of *E. multilocularis* and other gastrointestinal parasites in Swiss domestic cats and to compare the diagnostic sensitivity of different methods for the detection of intestinal taeniid infection.

**Methods:**

Faecal samples from 146 cats were included in the study. Faecal samples only were available from 55 cats; for the other 91 cats, necropsy was performed in addition to faecal sample testing. All (*n* = 146) faecal samples were analysed by a combined sedimentation/flotation technique (44% ZnCl_2_) and by the sodium acetate-acetic acid-formalin (SAF) sedimentation technique; when sufficient material was available (*n* = 121 samples) the Baermann-Wetzel technique was also used. Additionally, all samples were analysed by two coproantigen (copro)-quantitative PCRs (qPCR): (i) a multiplex qPCR able to detect and differentiate between *E. multilocularis*, *Echinococcus granulosus* sensu lato and *Taenia* spp./other cestodes (CEST-qPCR) and (ii) an *E. multilocularis*-specific qPCR (EM-qPCR). Finally, the intestines were examined macroscopically and microscopically for parasite stages at necropsy (*n* = 91) and using an intestinal scraping technique (IST) (*n* = 64).

**Results:**

Of the 146 cats examined, 24 (17.1%) were infected by intestinal parasites, namely *Hydatigera* (syn. *Taenia*) *taeniaeformis* (8.9%), *Toxocara cati* (6.1%), *Capillaria* sp. (3.4%), hookworms (3.4%), *Mesocestoides litteratus* (1.4%), *Giardia* sp. (1.4%), *Cystoisospora rivolta* (1.4%), *Cystoisospora felis* (0.7%), *Toxoplasma gondii* (0.7%), *Hammondia hammondi* (0.7%) and *Strongyloides* sp. (0.7%). Necropsy and the IST revealed adult *H. taeniaeformis* in 12 animals, of which eight faecal samples were positive by the CEST-qPCR (sensitivity = 67%) and six samples by the sedimentation/flotation technique (sensitivity = 50%). No *E. multilocularis* infection was detected in the sampled cats. Using Bayesian latent class analysis, the mean posterior prevalence probability was 0.0% (95% confidence interval 0–0.83%) for *E. multilocularis*.

**Conclusions:**

There was no evidence of *E. multilocularis* infection among the 146 cats examined, suggesting that the prevalence of this parasite is low (< 1%) in the Swiss domestic cat population. Nonetheless, some of the sampled cats were infected by parasites that have rodents as intermediate hosts, demonstrating successful predation by these cats, and some were infected with zoonotic parasites. Cats therefore should not be disregarded as potential hosts for *E. multilocularis* and other zoonotic parasites.

**Graphical Abstract:**

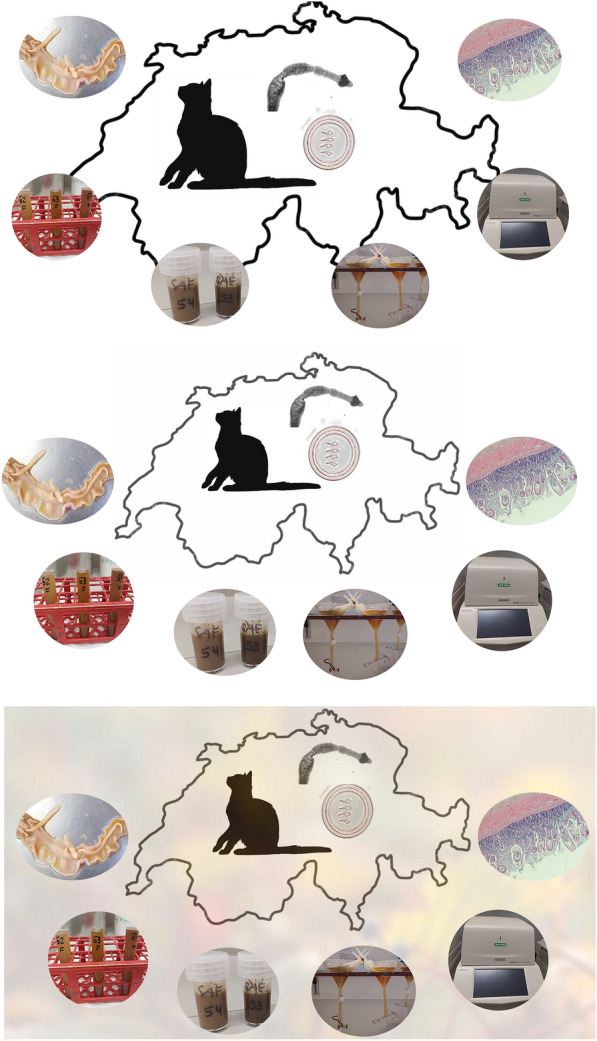

**Supplementary Information:**

The online version contains supplementary material available at 10.1186/s13071-023-05983-y.

## Introduction

In Switzerland, an estimated 1.8 million domestic pet cats live with a human population of approximately 8.6 million people, i.e. about 30% of all households have at least one cat [1]. Further, 100,000–300,000 stray cats roam freely in Switzerland [2]. This large cat population and the distinctive proximity of cats to humans, often including children and elderly people, make them a potential source of zoonotic pathogens, including parasites such as *Toxoplasma gondii* and *Toxocara cati*. The prevalence of these parasites in cats varies strongly depending on the geographical region of origin, frequency of veterinary care, lifestyle (stray, shelter, privately owned) and presence of intermediate and transport hosts.

*Echinococcus multilocularis* is a widely distributed parasite in the Northern hemisphere and is the cause of alveolar echinococcosis (AE) in humans, an emerging zoonotic disease [3, 4]. Foxes and other canids are the main definitive hosts of this parasite and responsible for environmental contamination with eggs [5–7]. Many rodent species serve as intermediate hosts [8, 9]. Additionally, a wide spectrum of mammal species can become accidental hosts and develop AE, such as humans, non-human primates, domestic pigs, Eurasian and Canadian beavers (*Castor fiber* and *C. canadensis*) and even domestic dogs [10–13]. Switzerland has been considered highly endemic for this parasite for decades, alongside other European countries, Russia, central Asia and western China [4, 14]**.** In Switzerland, the prevalence of *E*. *multilocularis* can reach up to 53% in the fox population, mainly in the northern and western parts [4] of the country, with a positive correlation between fox densities and the incidence of AE in humans [15]. Urban fox populations observed in many European countries lead to environmental contamination in the proximity of humans and their pets [16–18].

Comparative studies between foxes, dogs, raccoon dogs and cats suggest that cats play a much smaller role in the environmental contamination with *E. multilocularis* eggs than other definitive hosts. Cats showed a lower infection rate and shorter patent period, developed a lower worm burden and were estimated to have a lower biotic potential (total eggs excreted) than all other host species [6, 7]. On the other hand, cats can be a potential source of environmental contamination with *E. multilocularis* eggs in highly endemic areas [19]. Additionally, cat ownership, especially linked with unsupervised outdoor access of the cats, has been identified as a significant risk factor for human AE in case–control studies [20–22]. The majority of domestic cats in Switzerland are estimated to have unsupervised outdoor access [1]. Furthermore, even pet cats display a distinct predatory behaviour and will therefore occasionally prey on infected intermediate hosts, thus supporting the life-cycles of many parasites that affect not only felines but that can also play an important role in public health due to their zoonotic nature, such as *E. multilocularis*, *T. cati* and *T. gondii*.

Studies on *E. multilocularis* in cats generally report a very low prevalence in most western European countries. However, most studies utilized coprology as the detection method, which is well known to have a low sensitivity for taeniid eggs [23, 24]. Therefore, the aim of our study was to assess the prevalence of *E. multilocularis*, as well as of other zoonotic endoparasites, in domestic cats in Switzerland using different diagnostic approaches: routine coprological (copro) methods, copro-quantitative PCR (qPCR) techniques, the intestinal scraping technique (IST) followed by sedimentation and flotation of the flow-through and histopathology. Simultaneously, we compared the diagnostic performance of these various techniques for the detection of taeniid infections.

## Methods

### Sample collection

**Fig. 1:**
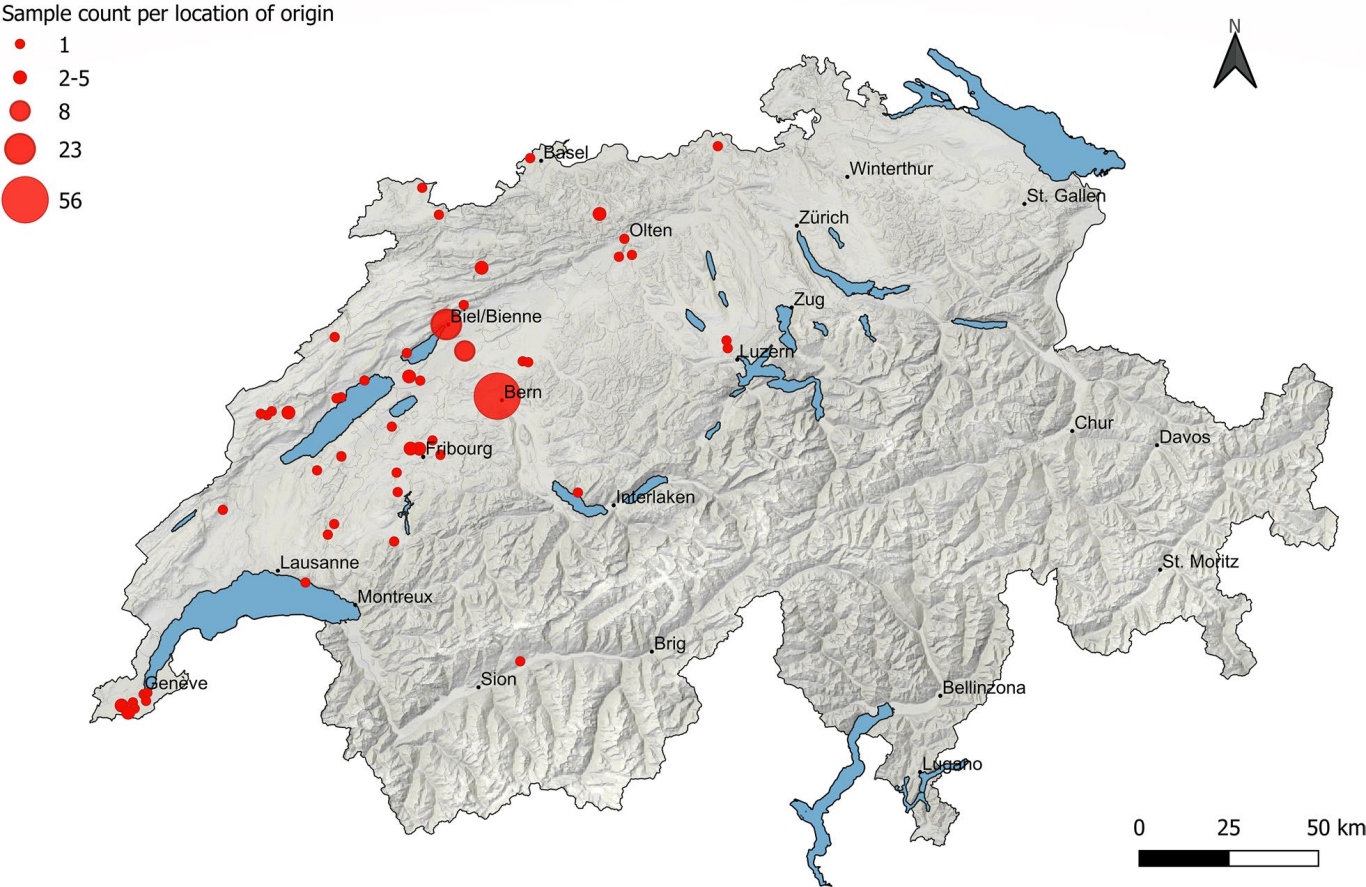
Map of Switzerland showing the geographical distribution of the 146 cat samples per location of origin

From March to September 2022, a total of 146 faecal samples were collected from privately owned cats, predominantly from the northwestern region of Switzerland (Fig. 1). Faecal samples or large intestine contents were obtained either from the routine diagnostic laboratory at the Institute of Parasitology, University of Bern (group 1, *n* = 55) or during necropsy (group 2, *n* = 91). The dead cats were disposed of at the Institute of Animal Pathology, University of Bern and kept frozen at − 20 °C until used for teaching and research purposes. Only cats older than 3 months of age were included in the study. Exact information on age, health status and outdoor access was not available for all animals.

### Routine coprology

Collected faecal samples or large intestine contents collected at necropsy were stored at 4 °C for a maximum of 3 days until analysis. All samples (*n* = 146) were analysed by: (i) the combined sedimentation/flotation technique using a 44% zinc chloride solution (specific density [S.D.] = 1.3) as flotation medium [25] and (ii) the sodium acetate-acetic acid-formalin (SAF)—concentration technique [25]. In addition, when sufficient material was available (*n* = 121), the Baermann–Wetzel technique was performed [25]. Parasite stages were photographed and measured using a digital image processing system (Nikon NIS-Elements—Microscope Imaging Software; Nikon Corp., Tokyo, Japan) and identified through morphological characterization [25]. *Isospora*-type oocysts were further differentiated into *Toxoplasma*-like and *Cystoisospora* species by their respective size [25], and *Toxoplasma*-like oocysts were further differentiated by PCR. Eggs of *Taenia* spp., *Hydatigera* spp., and *Echinococcus* spp. were grouped as taeniid eggs and further differentiated by PCR.

### Necropsy, histopathology and intestinal scraping technique

During necropsy, the intestines of 91 cats were cut open longitudinally and macroscopically examined for intestinal parasites. Detected nematodes and cestodes were stored for morphological and/or molecular characterization. For histopathological examination, sections of the duodenum and proximal jejunum were obtained [7], fixed in 4% neutral buffered formalin and embedded in paraffin. Multiple sections were prepared (thickness: 4 μm), mounted on glass slides and stained with haematoxylin and eosin (HE). Finally, the samples were analysed by light microscopy.

For 64 cats, the whole intestines were frozen at − 80 °C for at least 4 days. The IST was adapted from methods described by Hofer et al. [26]. Briefly, the intestinal mucous layer was scraped from the entire length of the small intestine using glass slides and transferred to a vessel containing 180 ml of 0.9% NaCl solution; the suspension was subsequently filtered applying a 3-step sieving technique, with filtration through a 2-mm, then a 1-mm and lastly a 250-μm mesh sieve. The remnants on each sieve were separately placed in a round plastic dish and analysed under a stereomicroscope (magnification 40×). The flow-through was allowed to sediment for 24 h, following which the supernatant was discarded, and the sediment was additionally investigated for parasite products by sucrose flotation (S.D. = 1.3).

### Molecular diagnostics

DNA extraction for the faecal/intestinal contents was performed using a commercial kit (Quick-DNA Fecal/Soil Microbe Miniprep Kit, Zymo Research Corp., Orange, CA, USA) according to the manufacturer’s instructions. DNA from adult stages of helminths that were found in the intestine during necropsy was extracted using the commercial DNeasy Blood & Tissue Kit (QIAGEN GmbH, Hilden, Germany) following the manufacturer's instructions.

#### CEST-quantitative PCR

All faecal/intestinal content samples were analysed with a specific TaqMan-probe-based multiplex real-time PCR (qPCR) able to simultaneously detect and distinguish between DNA from *E. multilocularis*,* Echinococcus granulosus* sensu lato (*E. granulosus* s.l.) and other cestodes (CEST-qPCR). This CEST-qPCR targeted mitochondrial genes for NADH dehydrogenase subunit 1 (*nad*1) and the small subunit of ribosomal RNA (*rrnS*) by using primers originally described by Trachsel et al. [27] for conventional PCR. The design of the probes was based on the use of the programme Primer3 Input, version 0.4.0 (https://bioinfo.ut.ee/primer3-0.4.0). For qPCR detection, TaqMan fluorescence probes were designed to discriminate among *E. multilocularis* (probe EMR-TAQ labelled with Cy5), *E. granulosus* (probe EGR-TAQ labelled with FAM) and other taeniids (probes CEST-TAQ 1–5 labelled with HEX) in a multiplex format (Table 1). Primers and TaqMan probes were purchased from Microsynth (Balgach, Switzerland). Reactions were performed in a final volume of 10 μl containing 5 μl of commercial master mix (SensiFAST™ Probe No-ROX Kit; Meridian Bioscience, Cincinnati, OH, USA), 0.5 μl of primer mix (containing 2 μM of primers CEST1, CEST2, CEST3, CEST4, and 16 μM of primer CEST5; Table 1), 0.1 μl detection probe mix containing seven probes (Table 1) at a concentration of 10 µM each, 0.4 µl of H_2_O and 4 μl of sample DNA. Positive (DNA extracts from *E. multilocularis*, *E. granulosus* and *Taenia saginata*) and negative (H_2_O) controls were used in each run. DNA amplification was performed in the CFX96 qPCR detection system (Bio-Rad Laboratories AG, Cressier, Switzerland) starting with 1 cycle of 95 °C for 5 min, followed by 50 cycles of 10 s at 95 °C and 30 s at 58 °C. PCR products were measured after each cycle by the detection of light emission from the different fluorophores at the end of the 58 °C incubation step. For analysis of the results, CFX manager software version 1.6 (BioRad Laboratories AG) was used. Reactions with a cycle threshold (Ct) of < 50 cycles were defined as positive.

**Table 1:** Primer and TaqMan probe sequences used in the CEST-quantitative PCR

Primer sequences^a^			
Target species	Gene	Primer designation	Sequence (5′–3′)
*Echinococcus multilocularis*	nad1	CEST1	5′-TGCTGATTTGTTAAAGTTAGTGATC-3
	nad1	CEST2	5′-CATAAATCAATGGAAACAACAACAAG-3′
*Echinococcus granulosus*	rrnS	CEST4	5′-GTTTTTGTGTGTTACATTAATAAGGGTG-3
	rrnS	CEST5	5′-GCGGTGTGTAC(A/C)TGAGCTAAAC-3′
*Taenia* spp.^c^	rrnS	CEST3	5'-(C/T)GA(C/T)TCTTTTTAGGGGAAGGTGTG-3'
	rrnS	CEST5	5′-GCGGTGTGTAC(A/C)TGAGCTAAAC-3′

TaqMan probe sequences^b^			
Target species	Probe designation	Sequence (5′-3′)	
*E. multilocularis*	EMR-TAQ	5′-(Cy5)-TTGTTGTGT(G/C)CTGGTTGGGGT-(BHQ-2)-3′	
*E. granulosus*	EGR-TAQ	5′-(FAM)-ATTTA5GACTT6ATA7TAATG-(BHQ-1)-3′ (5 = LNA-G, 6 = LNA-A, 7 = LNA-G)^d^	
*Taenia* spp.^c^	CEST1-TAQ	5′-(HEX)-TATGTTGGTGTATATCTGGTTTAATATT-(BHQ-1)-3′	
	CEST2-TAQ	5′-(HEX)-TAT(G/A)TTGGTGTATATCTGGTTTAATATT-(BHQ-1)-3′	
	CEST3-TAQ	5′-(HEX)-TATGTT(G/T)GTGTATATCTGGTT(G/T)AATATT-(BHQ-1)-3′	
	CEST4-TAQ	5′-(HEX)-TATGTTGGTGTATATCTGGTTTGATATT-(BHQ-1)-3′	
	CEST5-TAQ	5′-( HEX)-TATGTTGGTGTATATCTGATTTAATATT-(BHQ-1)-3′	

CEST-qPCR is a specific TaqMan-probe-based multiplex real-time PCR (qPCR) able to simultaneously detect and distinguish between DNA from *E. multilocularis*,* E. granulosus* sensu lato and other cestodes

^a^Trachsel et al. [27]

^b^Reference: this study

^c^*Taenia saginata, T. solium, T. serialis*, *T. crassiceps*, *T. pisiformis, T. ovis, T. polyacantha, T. multiceps*; also *Hydatigera* spp., *Versteria* spp., *Spirometra* spp., *Mesocestoides* spp., *Hymenolepis* spp., *Dipylidium caninum* and *Diphyllobothrium latum*

^d^Numbers indicate nucleotide positions containing locked nucleic acid (LNA) substitutions

#### *Echinococcus multilocularis*-specific qPCR

All faecal/intestinal content samples were analysed for the presence of *E. multilocularis* DNA based on the amplification of a part of the mitochondrial gene* rrnL* with a specific TaqMan-qPCR (ES-qPCR) in a CFX96 qPCR detection system (Bio-Rad Laboratories AG), as previously described [28]. The same general qPCR reagents previously mentioned for the CEST-qPCR were used. The detection limit of this qPCR has been reported as one egg of *E. multilocularis* in 0.5 g of faecal sample [28].

#### qPCR *Toxoplasma gondii*

Samples positive for *Toxoplasma*-like oocysts were further investigated with a TaqMan-based real-time PCR as previously described by Pardo Gil et al. [29] to confirm *T. gondii* oocysts and distinguish them from the morphologically similar oocysts of *Hammondia* spp.

#### Conventional PCR and sequencing

DNA extracted from cestode stages collected during necropsy, IST and intestinal content samples positive for *Taenia* spp. in the CEST-qPCR were analysed by a conventional multiplex-PCR as described by Trachsel et al. [27]. Samples with *Toxoplasma*-like oocysts, negative for *T. gondii,* were additionally investigated with a conventional PCR targeting the 18S ribosomal RNA (rRNA) fragment employing COC-1 and COC-2 primers [30]. Positive, amplified products were purified (DNA Clean & Concentrator-5; Zymo Research, Irvine, CA, USA) and sent for Sanger sequencing (Microsynth, Balgach, Switzerland). The obtained sequences were aligned with the Geneious Prime software (version 2023.0.4) and then compared with GenBank entries using NCBI BLAST.

### Statistical analysis

The observed prevalence and exact binomial 95% confidence intervals (CIs) were calculated for each parasite according to Clopper and Pearson [31], with the exception of *E. multilocularis*. For *E. multilocularis,* the confirmed EM-qPCR results were analysed in a Bayesian framework using latent class analysis as described by Branscum et al. [32]. We used peer-reviewed published literature available at the time to inform our priors and build the statistical model. We used beta prior distributions for the sensitivity (Se) and the specificity (Sp) of the EM-qPCR as follows:

$$\begin{aligned}&\mathrm{Se}\sim Beta\left({a}_{\mathrm{Se}},{b}_{\mathrm{Se}}\right)\\ & \mathrm{Sp}\sim Beta\left({a}_{\mathrm{Sp}},{b}_{\mathrm{Sp}}\right)\end{aligned}$$

These parameters were based on Knapp et al. [28] and were determined as follows: *a*_Se_ = 25, *b*_Se_ = 4, *a*_Sp_ = 26 and *b*_Sp_ = 2.

The infection prevalence *π* was modelled using a mixture distribution as follows:

$$\begin{aligned}& \pi \sim Beta\left({a}_{\pi },{b}_{\pi }\right)\,\,\,\mathrm{ with \, probability }\,\,\tau \\ & \pi =0\,\,\,\mathrm{ with \, probability }\,\,1-\tau\end{aligned}$$

where *τ* is the probability that the disease is present in the cat population. Again, the parameters of the prior distribution were based on published literature. Two studies assessed the prevalence of *E. multilocularis* in Swiss cats, one of which reported one positive sample of 263 tested (0.4%) [33] and the second reported zero prevalence [34]. Zero prevalence was also reported in three of seven European countries [35, 36]; the prior probability *τ* was therefore set to 0.5. In the available literature, the highest reported prevalence was 6% in Poland [48]; therefore, the prior prevalence parameters were set to *a*_*π*_ = 1 and *b*_*π*_ = 50, so that 95% of the prior probability density function fell below 6%.

The data analysis was performed with WinBUGS (version 1.4.3) [37] called from R (version 4.2.1) (www.cran.r-project.org) through the R2WinBUGS (version 2.1-21) [38] package. We run three chains for 10,000 iterations and discarded the first 500 as a burn-in and thinned to every fifth sampled value.

#### Sensitivity calculation of sedimentation/flotation and CEST-qPCR

The sensitivity of faecal flotation and the CEST-qPCR was estimated using post-mortem recovery of adult parasites as the gold standard. It was calculated as the ratio between the number of true positives correctly detected by the respective technique and the total number of true positives detected by necropsy.

## Results

Taking into consideration all applied methods (routine coprological methods, necropsy, IST, copro-qPCRs and histopathology), 25 of the 146 cats (17.1%; 95% CI 11.4–24.2) were positive for intestinal parasites, but none of the animals showed *E. multilocularis* infection (Table 2).

**Table 2:** Overview of total positive parasitological results obtained by coprological techniques, molecular methods, necropsy, and intestinal scraping technique in domestic cats

Group	Animal ID	Coprology	Faecal CEST-qPCR^a^	Necropsy (adult worms)	Intestinal scraping technique
1^b^ (*n* = 5)	S22_5915	*T. cati*	*Taenia* pos (*M. litteratus*^d^)	NA	NA
	22D1599	*Giardia* sp.	Neg	NA	NA
	22D1031	*T. gondii*^c^	Neg	NA	NA
	22D2759	*Giardia* sp.	Neg	NA	NA
	22D2520	*C. felis*	Neg	NA	NA
2^b^ (*n* = 20)	PVK2	*Capillaria* sp.	Neg	Neg	NA
	THK1	Taeniid eggs *T. cati*	*Taenia* pos	*H. taeniaeformis*^d^	NA
	PVK7	*T. cati*	Neg	*T. cati*	NA
	PVK11	Taeniid eggs *C. rivolta*	*Taenia* pos	*H. taeniaeformis*^d^	NA
	PVK13	Taeniid eggs *Capillaria* sp. Hookworm	*Taenia* pos	*H. taeniaeformis*^d^	NA
	PVK18	Neg	Neg	Neg	*M. litteratus*^d^ (5 immature worms)
	PVK20	Neg	Neg	Neg	*A. tubaeforme* (1 male)
	PVK23	Taeniid eggs	*Taenia* pos	*H. taeniaeformis*^d^	*H. hammondi*^d^
	PVK25	Hookworm *C. rivolta* *Strongyloides* sp.	*Taenia* pos	*H. taeniaeformis*^d^	*T. cati* (1 immature)
	PVK26	Taeniid eggs	*Taenia* pos	*H. taeniaeformis*^d^	*A. tubaeforme* (1 male) + *T. cati* (1 immature)
	S22_7062	Taeniid eggs	*Taenia* pos	*H. taeniaeformis*^d^	NA
	PVK32	*Capillaria* sp. *T. cati*	Neg	*T. cati*	Neg
	PVK42	*T. cati*	Neg	*T. cati*	Neg
	PVK45	*Capillaria* sp. Hookworm *T. cati*	Neg	*T. cati*	*T. cati* (1 immature)
	PVK57	Neg	*Taenia* pos	Neg	*H. taeniaeformis*^d^ (1 egg)
	S22_7953	Neg	Neg	*H. taeniaeformis*^d^ & *T. cati*	Neg
	PVK70	Neg	Neg	*H. taeniaeformis*^d^	Neg
	PVK71	Neg	Neg	*H. taeniaeformis*^d^	Neg
	S22_8189A	*Capillaria* sp.	Neg	*H. taeniaeformis*^d^	NA
	S22_8189B	Neg	*Taenia* pos	*H. taeniaeformis*^d^	NA
Positive (total)	25 (146)	18 (146)	10 (146)	16 (91)	7 (64)

*NA* Not available,* Neg* negative test result,* Pos* positive test result; *A. tubaeforme* = *Ancylostoma tubaeforme*; *C. felis* = *Cystoisospora felis*;* C. rivolta* = *Cystoisospora rivolta*;* H. hammondi* = *Hammondia hammondi*; *H. taeniaeformis = Hydatigera* (syn. *Taenia*)* taeniaeformis*;* M. litteratus* = *Mesocestoides litteratus*;* T. cati *= *Toxocara cati*; *T. gondii* = *Toxoplasma gondii*

^a^A specific TaqMan-probe-based multiplex quantitative PCR (qPCR) able to simultaneously detect and distinguish between DNA from *Echinococcus multilocularis*,* E. granulosus* sensu lato and other cestodes

^b^Group 1: faecal samples only. Group 2: necropsied cats

^c^Identification confirmed by specific qPCR

^d^Identification confirmed by conventional PCR and sequencing

### Coprological examination

With routine faecal coprology (i.e. combined sedimentation/flotation technique, sodium acetate-acetic acid-formalin—concentration technique and Baermann), 12.3% (18/146; 95% CI 7.5–18.8) of the samples tested positive for parasite eggs, oocysts, cysts and/or larvae. Taeniids and *T. cati* were the most frequently detected parasites, with a prevalence of 4.1% (6/146; 95% CI 1.5–8.7) each, followed in order of decreasing prevalence by *Capillaria* sp. (3.4% [5/146]; 95% CI 1.1–7.8), hookworms (2.1% [3/146]; 95% CI 0.4–5.8), *Giardia* sp. (1.4% [2/146]; 95% CI 0.2–4.8), *Cystoisospora rivolta *(1.4% [2/146] 95% CI 0.2–4.8), *Cystoisospora felis* (0.7% [1/146]; 95% CI 0–3.7), *T. gondii* (0.7% [1/146]; 95% CI 0–3.7) (confirmed by qPCR) and *Strongyloides* sp. (0.7% [1/146] 95% CI 0–3.7) (confirmed by morphological identification of third-stage larvae in coproculture) (Table 2). Taeniid eggs were only detected in samples from necropsied cats (6.6% [6/91]; 95% CI 2.5–13.8).

### Necropsy, histopathology and IST

Adult helminths were macroscopically visible during necropsy in 16/91 cats (17.6%; 95% CI 10.4–27.0) (Table 2). Histopathology revealed coccidian parasite stages in one cat, which was positive for *C. rivolta* oocysts by coprology. *Echinococcus* spp*.* infection was not detected by either macroscopical examination or histopathology in any of the 91 necropsied cats.

The IST and/or flotation of IST sediment in 64 cats allowed the detection of additional parasites in seven cases, i.e. a taeniid egg, *Mesocestoides litteratus* (immature worms), *Ancylostoma tubaeforme* (male worms), *T. cati* (immature worms) and *T. gondii*-like oocysts (further identified as *Hammondia hammondi* by PCR, see following text) (Table 2).

### Copro-qPCRs

The CEST-qPCR analyses of faecal/intestinal content detected 10/146 (6.9%; 95% CI 3.3–12.2) samples positive for DNA of *Taenia* spp./other cestodes (Table 2). No *E. multilocularis* or *E. granulosus* s.l. DNA was detected. The *E. multilocularis*-specific EM-qPCR analysis was negative for all 146 faecal/intestinal content samples.

### Conventional PCRs and sequencing

**Fig. 2:**
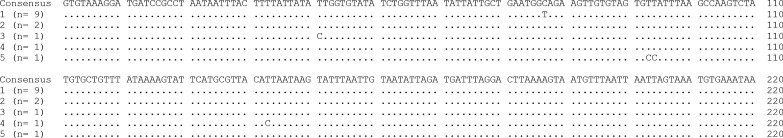
Identified haplotypes of *Hydatigera* (syn. *Taenia*) *taeniaeformis* of 14 samples from 12 domestic cats. Haplotypes: 1, OR268953–OR268961; 2, OR268952 and OR268962; 3–5, OR268963–OR268965

The conventional multiplex-PCR analysis identified 16 positive samples with primers CEST3-CEST5 (*Taenia* spp. and other cestodes). Fourteen of these sequences (220 bp, with trimmed primer regions) were obtained from 11 adult worms found in 10 cats (two of the worms were derived from the same cat), faeces of two cats and IST of one cat and were deposited in GenBank (Accession no. OR268952–OR268965). These sequences showed 99.1–100% identity with *Hydatigera taeniaeformis* sensu lato (including *Hydatigera kamiyai*) (GenBank ID JQ663994 and MN505198). Five different haplotypes were identified: one haplotype (1) was detected in nine of the samples (OR268953–OR268961), a second haplotype (2) was detected in two samples (OR268952 and OR268962, with one of the sequences [OR268952] being 38 bp shorter) and the other three haplotypes (3–5) (OR268963-OR268965) were observed in one sample each (Fig. 2). It is interesting to note that the two analysed worms from the same cat showed different haplotypes.

Two of the sequences (223 bp, with trimmed primer regions) obtained from a faecal sample and one adult worm had an identity of 99.6% and 100% with *M. litteratus* (GenBank ID MN505210 among others), respectively, and were submitted to GenBank (Accession no. OR268982–OR268983). Oocysts detected by flotation of IST sediment were identified as *H. hammondi* by COC1-COC2-PCR and sequencing (100% identity with GenBank AH008381, KT184369, KF854253).

### Diagnostic methods comparison

In 91 cats, three diagnostic methods were implemented for the detection of cestodes, including (i) macroscopic evaluation of the intestine during necropsy; (ii) faecal sedimentation-flotation; and (iii) molecular diagnostics with copro-qPCR. Infection with adult taeniids (i.e. *H. taeniaeformis*) was detected in 13.2% (95% CI 7.0–21.9) of these cats during necropsy (12/91), in 6.6% (95% CI 2.5–13.8) of cats with the flotation technique (6/91) and in 9.9% of cats (95% CI 4.6–17.5) in the CEST-qPCR analysis (9/91) (Table 3). All samples positive for taeniid eggs in faecal flotation were also positive in the CEST-qPCR analysis (6/6). From the six samples positive for taeniids in necropsy and negative in coprology, two were also positive by qPCR. One of the cats negative for taeniids in necropsy and faecal flotation tested positive in the CEST-qPCR; in the flotation test of IST sediment from this cat a single taeniid egg was observed. The sensitivity of the CEST-qPCR and the flotation technique were estimated to be 66.7% and 50%, respectively, when compared to the necropsy findings (Table 3).

**Table 3:** Comparison of diagnostic sensitivity of necropsy, coprology and faecal CEST-qPCR to detect cestode infection in Swiss cats (*n* = 91)

	Necropsy	Coprology	CEST-qPCR^a^
Positive samples (*n*)	12	6	9
Prevalence (%)	13.2	6.6	9.9
Estimated sensitivity (%)	100^b^	50	66.7^c^

^a^A specific TaqMan-probe-based multiplex quantitative PCR (qPCR) able to simultaneously detect and distinguish between DNA from *Echinococcus multilocularis*,* E. granulosus* sensu lato and other cestodes

^b^Necropsy was set as the gold standard

^c^One of the positive samples by CEST-qPCR was not included in this calculation because it was negative in necropsy. However, a taeniid egg was detected by IST-sediment flotation (Table 2: Animal ID: PVK57)

### Prevalence of infections considering all applied methods

Overall, considering all applied methods, the most frequently found parasite was *H. taeniaeformis* (13/146 cats [8.9%]; 95% CI 4.8–14.7), followed by *T. cati* (9/146 cats [6.1%]; 95% CI 2.9–11.4) (Table 2). *Capillaria* sp. was found in 5/146 cats (3.4%; 95% CI 1.1–7.8), hookworms in 5/146 cats (3.4%; 95% CI 1.1–7.8), *M. litteratus* in 2/146 cats (1.4%; 95% CI 0.2–4.8), *Giardia* sp. in 2/146 cats (1.4%; 95% CI 0.2–4.8), *C. rivolta* in 2/146 cats (1.4%; 95% CI 0.2–4.8), *C. felis* in one cat (0.7%; 95% CI 0–3.7), *T. gondii* in one cat (0.7%; 95% CI 0–3.7), *H. hammondi* in one cat (0.7%; 95% CI 0–3.7) and *Strongyloides* sp. in one cat (0.7%; 95% CI 0–3.7). Infection with helminths (21/146 [14.4%]; 95% CI 9.1–21.1) was more prevalent than infection with protozoans (7/146 [4.8%]; 95% CI 1.5–8.7) (Table 2).

### Prevalence of co-infections

Cats found to be infected with a single gastrointestinal parasite (14/146 [9.6%]; 95% CI 5.3–15.6) were more numerous than cats co-infected with two parasite species (7/146 [4.8%]; 95% CI 1.5–8.7) or three different parasite species (3/146 [2%]; 95% CI 0.4–5.9). One cat (ID PVK25) was even co-infected with five parasites (Table 2).

### Prevalence of* E. multilocularis*

No *E. multilocularis* was detected in this study. The most likely prevalence was 0%, with a median and mean of the posterior density function of 0.0% and 0.12%, respectively, and a 95% upper credible limit of 0.83%. To check the influence of the prior information on the posterior prevalence, we re-ran the model, varying the prevalence beta priors and the *τ* parameters. Overall, the results were robust against prior changes. Using flat prior for the prevalence led to lower posterior prevalence values, whereas using flat prior and simultaneously increasing prior on *τ* to ≥ 0.99 led to higher posterior prevalence estimates. The posterior prevalence estimation (%) after varying model priors is shown in Additional file 1: Table S1.

## Discussion

The results of the present study provide an overview of the status of gastrointestinal parasite infections in the Swiss domestic cat population, with a focus on cestode infections and a bias for the north-western regions of Switzerland. These regions historically have a high prevalence of *E. multilocularis* in foxes [4]. Only a few studies have focused on *E. multilocularis* prevalence in domestic cats in Switzerland: Zottler et al. [34] showed negative results for *E. multilocularis* in cats, while Deplazes et al. [33] found 1 of 263 cats (0.4%) to be positive for the parasite after necropsy.

A reliable diagnosis of *E. multilocularis* infection in definitive hosts can only be achieved post-mortem (i.e. sedimentation and counting technique [SCT], IST and shaking in a vessel technique [SVT] [39–41]), which is a major limitation for studies in pet canids and felids. Arecoline purgation was widely used in the past, but the controversial sensitivity [42, 43] and animal welfare issues linked to this technique have led to the abandonment of the method in most countries. Different coproantigen enzyme-linked immunosorbent assays (ELISAs) have been developed and tested for the detection of *E. multilocularis* infection in foxes, dogs and cats, and although they showed promising results, they are not widely used [33, 44]. Several copro-PCRs have been developed for the identification of *E. multilocularis*, which could present a good solution for monitoring *E. multilocularis* in both domestic and wild carnivores [24, 27, 28, 45]. Nevertheless, the diagnostic sensitivity of these techniques may be negatively affected in cats, in which this parasite has been reported to exhibit a low biotic potential [6]. Therefore, we used both a multiplex qPCR (based on Trachsel et al. [27]), which additionally allowed for detection of other cestode species, and a highly sensitive and specific qPCR for *E. multilocularis* [28].

Despite our considerable effort in using various diagnostic techniques, no *E. multilocularis* infection was detected in our sampled population. The prevalence estimation of *E. multilocularis* of 0% with an upper credible limit of 0.83% in cats in Switzerland, obtained by the Bayesian latent class analysis in the present study, is in accordance with previous findings reporting the prevalence of *E. multilocularis* in cats in Europe of between 0% and 0.38% [33, 34, 36]. However, one study from Poland reported a 6% prevalence of *E. multilocularis* in cats [48], and several copro-PCR studies on cat faecal samples collected in rural areas and kitchen gardens in France reported high prevalences of 1.2–9.3% [19, 35, 46, 47]. In these latter studies, deposition of multiple faecal samples by the same infected cats cannot be excluded; therefore, we did not include them in the priors for the Bayesian latent class analysis.

Taking the entire Swiss cat population into consideration (1.8–2.1 million cats [1, 2]), we could expect that up to 21,000 cats might be infected. The high densities of these pets and their common access to intermediate hosts can outweigh the low infection rates. Hegglin et al. [49] provided evidence showing that the total contribution of cats to environmental contamination with *E. multilocularis* eggs is < 0.2%, with other carnivores being responsible for the rest [49]. Nevertheless, in high-endemic areas, cats were shown to be a source of local environmental contamination [19]. Bastien et al. [47] showed that the density of cat faeces was significantly higher within kitchen gardens than outside them, while there was not significant difference in the distribution of fox faeces. These data indicate that cats from highly endemic areas could represent a source of contamination for human households. Therefore, even if cats rarely excrete eggs and are to be considered of lower epidemiological significance, in highly endemic areas [50], a higher deworming frequency of cats with unsupervised outdoor access and/or in contact with children, elderly and immunosuppressed persons is recommended.

In this study, the overall (17.1%; 95% CI 11.4–24.2) and coprological (12.3%; 95% CI 7.4–18.8) parasite infection rates were similar to those observed by Zottler et al. [34] in privately owned cats in Switzerland (11.7%). However, the latter study showed a markedly higher prevalence in stray cats (77.4%) and cats from shelters (21.8%), results that were confirmed by other studies [34, 51]. The latter cats are at a higher risk of parasite infection because of their dependence on hunting and scavenging for food, which exposes them to contaminated environments and intermediate and paratenic hosts; in addition, they usually do not receive anthelmintic treatments.

The results of the present study showed that mono-infections were more prevalent than co-infections with ≥ 2 intestinal parasites and that helminths were detected more frequently than protozoans, confirming the outcomes of other studies [34, 52, 53]. However, it is noteworthy that almost half of the positive cats (11/25) were co-infected by ≥ 2 intestinal parasites. Several intestinal parasites were found, including *H. taeniaeformis*,* T. cati*, *Capillaria* sp., *A. tubaeforme*, *M. litteratus*, *Giardia* sp*.*, *C. felis*, *C. rivolta*,* Strongyloides* sp., *T. gondii* and *H. hammondi*, some of which are of potential zoonotic concern. The most frequently detected parasites in our study were *H. taeniaeformis* and *T. cati*.

In this study, all detected taeniid worms and eggs were identified as *H. taeniaeformis*, which was confirmed by molecular methods. *Hydatigera taeniaeformis* infection in cats is exclusively the result of predation on small mammals. Results from necropsy revealed a higher prevalence of *H. taeniaeformis* (13.2%, 12/91) than did copro-qPCR (9.9%, 9/91) and coprology (6.6%, 6/91) of the same samples, showing that the copro-qPCR was more sensitive than the coprological examination for taeniid infections. This result is in line with those of previous studies [24, 39, 43]. Coprology could lead to an underestimation of infection prevalence, especially when using only flotation techniques. Possible causes of this apparent lower sensitivity are the presence of immature worms, a low number of adult worms and intermittent egg shedding or shedding of a low number of eggs. This observation could be extrapolated to infections with other taeniid species, such as *E. multilocularis* infection in cats, with this parasite showing a lower biotic potential (total eggs excreted) in cats than in other host species [6, 7]. The overall prevalence in our study was higher than that observed by necropsy alone because in one of the samples no adult parasites were detected, only a single egg by the IST and confirmed by PCR. This result could be due to the expulsion of adults after treatment or the intestinal passage of eggs. Interestingly, five different haplotypes of *H. taeniaeformis* were identified, with one of the haplotypes being overrepresented (Fig. 2). Noteworthy, one of the cats harboured worms corresponding to two different haplotypes, suggesting a high genetic variability of this parasite. In addition, it is important to mention that rare cases of human infection with *H. taeniaeformis* have been reported [54].

Neither *Dipylidium caninum* nor *Diphyllobothrium latum,* two non-taeniid tapeworms, were detected in this study. The absence of these parasites is not surprising, considering that recent studies have indicated a low prevalence of these parasites in the Swiss cat population [34, 59]. Therefore, detecting them within a small sample size is less likely; their DNA would, however, have been detected in the CEST-qPCR [27].

*Toxocara cati* was detected in 9/146 cats (6.2%), which is in line with the prevalence reported in other studies from Europe, ranging from 4.7% to 35% [34, 51–53, 55]. The relatively low prevalence observed in this study may be associated with the fact that only privately owned cats were analysed and that kittens aged < 3 months were excluded. Toxocariasis is an important zoonosis that can cause dermatological, visceral, neural and ocular disorders [56]. Also, it would appear that *T. cati* plays a more important role in environmental contamination and the spread of human toxocariasis than previously thought [57].

Hookworms are zoonotic nematodes with a worldwide impact on human health, causing cutaneous larva migrans (CLM) and “eosinophilic enteritis” [58], among other pathologies. We detected hookworm infection in 5/146 (3.6%) cats by either coprology (*n* = 3) or the IST (*n* = 2; only male *A. tubaeforme* worms). This prevalence is among the highest in Europe; however, previous studies relied on coprology alone [34, 51, 52, 55, 59, 60]. No further identification of hookworm and *Capillaria* sp. eggs found in coprology was attempted and, therefore, the risk of these parasite stages being present due to intestinal passage (feed contamination, preyed rodents, or birds) cannot be ruled out.

*Strongyloides* species that have previously been reported in cats include *S. felis*,* S. planiceps*,* S. tumefaciens* and *S. stercoralis.* Little data on the prevalence of these parasites are available to date, but cats have been shown to harbour the potentially zoonotic *S. stercoralis* and to develop similar pathological lesions as humans, raising the question of whether the parasite could be of importance in a one-health context [61]. *Strongyloides* sp. larvae were detected in one cat in the present study, but unfortunately the attempt at molecular identification failed.

The epidemiological role of domestic cats in the spread of zoonotic *Giardia* species is still debated; however, cats have been shown both to have a similar rate of *G. cati* and *G. duodenalis* infection as dogs and also to harbour a greater proportion of *G. duodenalis* than dogs [62]. The reported prevalence for *Giardia* spp. in cats in Europe has a very wide range from 0.7% to 12.6%. Barutzki et al. [60] reported that cats aged < 3 months and between 3 and 6 months showed significantly higher infection rates with *Giardia* spp. than did older cats. In this context, our exclusion of animals aged < 3 months from the study could explain the rather low prevalence of *Giardia* spp. detected in our study. In addition, the freezing of cats before necropsy and sample collection may also have influenced our results since *Giardia* was not detected in any of the 91 analysed intestinal contents.

Felids are the only hosts that can excrete *T. gondii* and *H. hammondi* oocysts and thus are responsible for environmental contamination [63]. Toxoplasmosis can lead to major health problems in immunocompromised patients and can have fatal consequences for the foetus if seronegative mothers are infected during pregnancy [64]. *Hammondia hammondi* is not a zoonotic protozoan parasite, but since its oocysts are not distinguishable from those of *T. gondii* by microscopical examination, molecular methods are required for the differential diagnosis. Our result of 0.7% prevalence for both *T. gondii* and *H. hammondi* nicely falls within the range previously reported in Switzerland (0.4 and 0.6%, respectively) [34, 65].

## Conclusion

Despite an intensive search, no intestinal infection with *E. multilocularis* was detected in any of the 146 cats included in this study. However, at least 10% (15/146) of the cats in our sample harboured *H. taeniaeformis*, *M. litteratus* and/or *H. hammondi*, suggesting that they successfully preyed on small mammals that can be natural intermediate hosts for *E. multilocularis*. Because of the relatively small sample size, we cannot rule out the possibility that cats may excrete *E. multilocularis* eggs at a low prevalence (< 0.83%) and thus contribute to household and environmental contamination. Future studies should be done on a larger sample size to address this possibility.

### Supplementary Information

**Additional file 1. Table S1**: Posterior prevalence estimation (%) after varying model priors in the Bayesian latent class analysis.

## Data Availability

All relevant raw data are given in the manuscript and Additional file 1, and sequences have been deposited in GenBank (Accession no. OR268952–OR268965 and OR268982–OR268983).
