# Supplementary material for: What is the role of Swiss domestic cats in environmental contamination with Echinococcus multilocularis eggs?
Source: Parasit Vectors. 2023 Oct 9;16:353. doi: 10.1186/s13071-023-05983-y (PMC10561489; doi:10.1186/s13071-023-05983-y)
Supplement: Supplementary file 1 — Additional file 1. Table S1: Posterior prevalence estimation (%) after varying model priors in the Bayesian latent class analysis. [file 13071_2023_5983_MOESM1_ESM.docx]

Additional file 1 Posterior prevalence estimation (%) after varying model priors

| Change in priors | median | mean | lower credible limit | upper credible limit |
| --- | --- | --- | --- | --- |
| None | 0.00 | 0.12 | 0.00 | 0.83 |
| uniform prevalence prior | 0.00 | <0.01 | 0.00 | 0.00 |
| τ = 0.9 | 0.20 | 0.41 | 0.00 | 1.55 |
| uniform prevalence prior and τ =0.9 | 0.00 | 0.05 | 0.00 | 0.26 |
| uniform prevalence prior and τ =0.99 | 0.00 | 0.35 | 0.00 | 1.69 |
| uniform prevalence prior and τ =1 | 0.55 | 0.80 | <0.01 | 2.41 |
